# Supplementary material for: Are carbon emissions trading and green financial instruments synergistic? -Comprehensive quantitative research based on content analysis
Source: PLoS One. 2024 Mar 7;19(3):e0298601. doi: 10.1371/journal.pone.0298601 (PMC10919601; doi:10.1371/journal.pone.0298601)
Supplement: S1 Data — (ZIP) [file pone.0298601.s001.zip › Data/地级市Z_F工作报告.zip!/║╙▒▒/╨╧╠¿╩╨/└┤╫╘╣1⁄2╓┌║┼í╛┬φ┐╦╩2╛▌═°í┐_2004.docx]

各位代表：

现在，我代表邢台市人民政府向大会作工作报告，请予审议。请市政协各位委员和列席会议的同志提出意见。

2003年工作回顾

　　2003年是我市发展进程中重要而又很不寻常的一年。全市人民在省委、省政府和中共邢台市委的领导下，以邓小平理论和“三个代表”重要思想为指导，认真贯彻党的十六大精神和中央及省重大决策部署，战胜“非典”疫情的巨大冲击，圆满完成市十二届人大一次会议确定的目标任务，在全面建设小康社会道路上迈出坚实的一步。

（一）经济建设呈现快速发展的好势头

——国民经济快速增长。全市国内生产总值完成505亿元，同比增长12.1%，超计划1.1个百分点。其中，一产77.9亿元，增长4.3%；二产288.5亿元，增长14.5%；三产138.6亿元，增长12%，是五年来经济增速最快的一年。全年有6项经济指标增幅在全省位次前移：国内生产总值由第8位升至第4位；固定资产投资由第5位升至第4位；规模以上工业增加值由第10位升至第8位；消费品零售总额由第7位升至第5位；地方一般预算收入由第10位升至第6位；出口总值由第4位升至第2位。

——农业经济稳步发展。粮食总产341.2万吨，与去年基本持平。棉花总产12.2万吨,增长16%。农林复合经营、优质专用小麦和专用玉米等面积明显增加。畜牧业总产值占农业总产值的比重达到37.1%。农业产业化经营率达到55.8%，提高1个百分点。全年农田水利基本建设投资2.8亿元，退耕还林36.1万亩，发展节水灌溉22.6万亩，解决了220个村、11.6万人的饮水困难。南水北调中线工程邢台段完成大沙河倒虹吸“四通一平”等前期工作，青山水库枢纽工程通过国家立项评估。全市建立了市级农业信息中心和农产品质量检测中心，17个县市建立了农业信息中心。农业开发继续保持全省领先地位。

——工业运行态势良好。全部工业增加值完成261.5亿元,增长14.5%，占国内生产总值的比重达51.8%，比上年增加4.1个百分点；规模以上工业实现利税47.3亿元，增长36.3%；工业经济效益综合指数达到152.1%，提高17.7个百分点。企业信息化、电子商务和电子政务有了新发展，入统工业高新技术产业实现增加值9.1亿元，增长12.4%。运用高新技术和先进适用技术改造提升传统产业，全年完成技改投资36.4亿元,增长16%。投资3.6亿元的邢钢烧结机及粉尘治理改造和5号高炉项目,投资1.2亿元的龙海公司炼铁及炼钢技改项目和投资1亿元的邢矿集团选煤厂改造等项目已相继投产达效。

——服务业发展较快。全年完成社会消费品零售总额156.3亿元，增长11.3%。家乐园超市、华业通讯、珂琳日化、华丰食品等一批连锁企业发展迅速，以糖酒、石油、烟草公司为龙头的三大配送体系和三级配送网络初步形成。加强市场管理和监控，整顿和规范了市场经济秩序。积极推进粮食体制改革，有7个县（市、区）顺利退出保护价。加大了旅游资源开发力度，全年完成项目投资1.3亿元，接待国内外游客170万人次，旅游业总收入达8亿元。太行奇峡群、扁鹊庙、前南峪、天梯山景区山迈入国家A级景区行列，崆山白云洞已完成4A级景区初评。

——财税工作取得新成绩。进一步深化财政体制改革，积极落实财税政策、惠企政策和惠农政策，大力培植财源，强化税收征管，全年全部财政收入完成35.5亿元，增长15.5%，创六年来最好水平。地方一般预算收入完成14.9亿元，增长19.4%；市本级收入完成14.06亿元，增长10.4%。全市20个县（市、区）的全部财政收入均实现超收，有7个县（市）收入超亿元。筹措资金1.2亿元，确保了防治“非典”的支出需要。争取转移支付资金8.2亿元，确保了公教人员工资及时发放和水平的提高。金融工作，进一步优化信贷结构，提高服务质量，有力支持了经济发展。2003年底，全市金融机构各项存款余额529.2亿元、贷款余额352.1亿元，分别比年初增加62.3亿元和25.3亿元。

（二）五项重点工作实现新突破

——民营经济进一步发展壮大。全年完成增加值393亿元，增长18%；上缴税金15.3亿元，增长20%，实现了速度效益同步增长。完成固定资产投入92亿元，增长30%。在1580个新上项目中，投资1000万元以上项目261个，亿元以上项目33个（比上年多27个），大项目投入占总投资额的比重达80%以上。特色产业成为民营经济的可靠支撑，所占比重达到60%以上；新建省级私营企业集团27家，累计达到60家，占全省三分之一；新建工业园区8个，完善提高31个，有6个被省政府列为重点扶持园区；省政府重点扶持的百家规模企业中，我市占9家。以华龙集团为核心的方便食品产业年产量超过100万吨，成为世界最大的方便面生产基地；羊绒产业销售收入突破130亿元，清河“羊绒之都”地位更加巩固。

——重点项目建设成绩显著。固定资产投资和重点项目建设呈现“一大两多”特点。“一大”，即投资规模大。全社会固定资产投资完成217.6亿元，增长29%。131个重点项目完成投资96.9亿元，增长189%，创历史最好水平。其中，14个省级重点建设项目完成投资27.1亿元，占年计划的181.5%，增长182.4%；117个市级重点建设项目完成投资69.8亿元，占年计划的275.4%，增长134.1%。各县市区已全部完成年度计划，其中高开区、邢台县、柏乡、桥东、沙河、宁晋、巨鹿、平乡、桥西等9个县市区超额完成全年任务的150%以上。“两多”，即大项目多和新开工项目多。投资5000万元以上的重点项目79个，比上年增加44个；投资亿元以上的46个，比上年增加27个。特别是兴泰发电2×30万千瓦供热机组、宁晋纺织城、邢临高速等投资20亿元以上项目的开工建设，改写了我市无特大型项目的历史。全年新开工重点项目80个，增长3.3倍。

——国企改革迈出新步伐。全市有830家国有企业进行了产权制度改革，改制面达90.6%。有19家中型国有公司制企业初步建立了现代企业制度。邢钢、邢矿、邢机、兴泰、红星5家省属企业分离办社会职能进展顺利，所属13所学校已全部移交地方管理。通过大力推进股权结构调整和破产重组，全市较为规范的股份制企业达546家，融资19.3亿元，盘活存量资产17.04亿元，有效地激发了企业活力。认真贯彻《中小企业促进法》，建立完善信用担保制度，培育了一批质量信用示范企业。

——开放工作取得新进展。全年利用国内市外资金32亿元，增长10%；实际利用外资5784万美元，增长22.3%。外贸出口高速增长，全年出口总额达3.02亿美元，增长55.8%。羊绒、单晶硅、服装等特色产品出口规模进一步扩大。全市出口额超100万美元企业增加到44家，增长52%；完成对外劳务合同980万美元、营业额1380万美元，分别增长50%和138%。

——城市和小城镇建设实现了新突破。全年完成城建总投资33亿元,比上年增加106%。其中,市政基础设施投资完成14亿元，增长27.3%。在市区，组织实施了牛尾河治理、车站南路新建、丰仓街改造、北二环路东延、公园东街北延、城市“四化”等一大批完善市区路网、拓展城市空间、改善城市生态环境的重点工程,提升了城市品位和载体功能。新增公共绿地面积32万平方米；新增燃气用户1.8万户、供热面积60.2万平方米，使市区燃气普及率和集中供热普及率分别达到80%和60%。进一步深化了城市管理体制改革，形成了一级政府、一级事权、一级财政、一级监督的环境卫生管理新格局。加大了城市综合整治力度，新建、改建农贸市场3个，拆除了影响市容市貌的各类违章建筑，取缔了市区违规三轮车，提高了城市管理水平。高开区扩区工作取得新成绩。同时，各县市区投资24亿元，新扩建了一大批道路、排水、垃圾处理、住宅小区等基础设施项目，加快了城镇化进程。全市城镇化水平达33.3%，提高2个百分点。沙河市恒利庄园荣获首届河北人居环境奖和全国人居环境范例奖。交通建设，谋划了今后五年交通基础设施建设规划，全年完成交通基础设施投入18.7亿元，是上年的3.5倍。邢临、青银两条高速公路开工建设，分别完成投资4亿元和8.2亿元，是年计划的333%和164%。新建农村公路572公里，新增通油路村216个,分别是年计划的195%和540%。

（三）各项社会事业全面协调发展

——切实加强科技和教育工作，增强了经济加速发展的支撑力。组织实施市级以上科技项目96项，完成重大新科技推广项目35个，实现高新技术技工贸收入35亿元、民营科技企业技工贸收入43亿元。全年引进各类人才768人，其中高级人才555人。“普九”工作成绩显著，广宗、柏乡、宁晋、威县通过省复查验收。积极发展高中段教育，全市万人均普通高中在校生达到186人。投资2972万元，改造中小学校舍7.6万平方米。教育改革和结构调整取得新成就，职业教育档次不断提高，河北机电学校升格为河北机电职业技术学院，“两本三专”高等教育框架建设迈出新步伐。

——认真抓好卫生和体育工作，增强了公众健康的保障能力。按照公共卫生建设的目标，投入1.2亿元建立了市县乡三级疫情预防控制网络，以大病统筹为主的新型农村合作医疗制度试点开始起步，城市社区卫生服务发展迅速，爱国卫生运动和全民健身活动成效显著。在省第五届农民运动会上，我市取得了5金、13银、8铜的好成绩，奖牌总数位居全省第三名。

——坚持人口、资源和环境协调发展，增强了可持续发展能力。以农村人口和城市流动人口为重点，认真开展计划生育，稳定低生育水平，人口自然增长率为4.3‰，低于年计划2.7个千分点。治理整顿国土资源管理秩序，保证了土地资源合理开发利用。建筑节能力度进一步加大，水和大气污染治理初见成效，市区空气质量二级和好于二级以上天数,比上年增加29天。新增造林面积49.2万亩，治理水土流失面积160平方公里，生态环境进一步改善。

——精神文明建设继续深入，民主法制建设进一步加强。加强了以社会公德、职业道德、家庭美德为主要内容的公民道德建设，深入开展民主评议行风活动，全力推进以“诚信邢台”为重点的精神文明创建活动，涌现出一批文明行业、文明单位、文明村镇和文明社区，社会文明程度进一步提高。内邱县被国家民政部命名为“全国村民自治模范县”。文艺创作和群众文化进一步繁荣，文化基础设施建设得到加强，彩色周末及假日文化活动蓬勃开展。以“双三好”为载体的双拥共建活动不断深入，进一步密切了军政军民关系。依法管理宗教事务，民族乡村发展步伐明显加快。自觉接受人大、政协监督，认真办理人大代表建议和政协委员提案，扎实推进政务、厂务、村务、校务公开，民主政治建设得到加强。积极推进“四五”普法，坚持依法行政，提高了政府工作的法制化、规范化和制度化水平。认真接待人民群众来信来访，努力排查调处民间纠纷，切实加强安全生产，强化社会治安综合治理，严厉打击各类违法犯罪活动，有力地维护了社会的安定团结。统计、外事、侨务、民兵、预备役、妇女、儿童、老龄、残疾人、审计、监察、广播电视、地震、气象、档案、人防、地方志、无线电管理等工作，也都取得了新成绩。

（四）人民生活有了新的改善

——城乡人民群众竦礁嗍祷荨３钦蚓用袢司芍涫杖?125元，增长8.5%；农民人均纯收入2728元，增长5.2%。进一步深化了农村税费改革，通过“减人、减事、减机构”，全年减轻农民负担5221万元。城乡居民储蓄存款余额年底达417.3亿元，比年初增长12.3%。收入的增加促进了人民群众生活质量的提高，城镇居民用于交通通讯、旅游保健和教育文化娱乐等方面的人均支出，分别增长59.5%、24.3%和15%。城镇居民人均住房使用面积达到18.8平方米，农村居民人均住房面积达到27.6平方米，人们的衣食住行有了明显改善。固定电话和移动电话用户分别增长8 % 和30%。

——困难群众的生产生活问题得到较好解决。积极落实再就业政策，加大投入，强化服务，全市新增就业人数3.1万人，安排5600名下岗职工再就业，为2199名“4050”人员提供了再就业援助，全市失业率低于省定目标0.5个百分点。下岗职工生活保障金、企业离退休人员养老金做到了按时足额发放。全市20个县（市、区）全部实行了医疗保险制度。企业养老保险、机关事业单位养老保险、失业保险和医疗保险扩面，分别完成全年任务目标的173%、102%、119.7%和114.9%。发放低保金3253万元，累计保障人数66.1万人。发放救灾款887万元，对1.88万户、2.9万农村特困群众实行定期定量救济。全年投入扶贫资金2900万元，重点扶持了350个国家和省扶贫开发重点村，使3.4万人脱贫。同时，以解决贫困人口温饱、增加贫困农民收入为中心，扶贫开发取得了明显成效。为贫困村打井79眼，配套39眼，修防渗管道12万米，修路50公里。安排生产生活条件恶劣的9个村、227户、766人进行了移民搬迁。

（五）抗击非典取得阶段性重大胜利

去年四月中旬，非典疫情突如其来。面对这场严峻考验，我们坚决贯彻执行省委、省政府的决策部署，在全省较早地建立了防护、监测、救治“三大体系”，采取了控制传染源、切断传染链、保护易感人群“三条措施”，率先出台免费治疗、定点补助、奖励首报“三项政策”，组织实施了遭遇战、攻坚战和成果保卫战“三大战役”，以“特殊的作风、特殊的效率、特殊的措施”成功地控制疫情扩散，使全市百万人口发病率始终在全省保持最低，夺取了这场斗争的阶段性重大胜利。在安与危、生与死的重大关头，邢襄儿女万众一心、众志成城；各级政府心系民生,处变不惊,做到了运转高效,指挥得当;广大干部和医务工作者，以身作则，奋不顾身，以实际行动书写了对党和人民的忠诚;公安干警和交通职工风餐露宿,封堵排查，杜绝了病源的传播;广大宣传工作者不畏风险,深入一线,营造了良好的“抗非”氛围。在这场斗争中，涌现出了一大批可歌可泣的先进模范人物和动人事迹，形成了“齐心协力、艰苦拼搏、无私奉献、敢于胜利”的“抗非”精神，已成为全市人民的宝贵精神财富和推进跨越式发展的强大动力！

总结去年的工作经验，可以概括为“五个必须”：必须坚持以邓小平理论和“三个代表”重要思想为指导，大力弘扬“树正气、讲团结、求发展”主旋律；必须高举发展大旗，始终把跨越式发展牢牢抓在手上；必须坚持与时俱进，破除影响邢台发展的思想和体制“两大障碍”；必须保持安定团结的政治局面，为跨越式发展提供有力保证；必须解决经济发展中的主要矛盾和薄弱环节，以重点突破带动和活跃全局。上述成绩的取得，是省委、省政府和中共邢台市委正确领导的结果，是全市人民共同努力的结果！在此，我代表市人民政府，向广大工人、农民、知识分子、各级干部和社会各阶层的建设者，向人大代表、政协委员，向各民主党派、工商联、无党派爱国人士和人民团体，向驻邢人民解放军、武警官兵、政法干警和民兵预备役人员，向省部属驻邢各单位，向关心支持我市发展的港澳台同胞、海外侨胞、国内外朋友，致以崇高的敬意和衷心的感谢！

在肯定成绩的同时，我们也清醒地看到我市面临的困难和差距：一是经济总量小。去年我市虽然经济发展速度较快，但这是在全国和全省都在高速发展的背景下取得的。目前我市GDP总量在全省处于第7位，人均排第9位；社会人均财政收入在全省处于末位。二是经济结构不尽合理。在三项产业中，一产比重较大，三产比重较小；在三产中，传统服务业多，新兴现代服务业少；在产品结构中，初级产品多，高科技含量、高附加值、高创汇产品少。同时，资本结构和组织结构不尽合理，企业设备利用率低。三是市属国有企业效益差。全年市属国有企业增加值增速比省属以上低7.8个百分点，比县市区属企业低19个百分点。在56户入统市属企业中，亏损企业达30户，亏损1.1亿元，占全部工业亏损额的88.4%。四是农民增收困难。由于农业结构调整步伐不快，农民增收渠道窄，尽管农民人均纯收入出现恢复性增长，但城乡差距仍在扩大，全年农民人均纯收入增速比城镇居民人均可支配收入低3.3个百分点。五是财政收支矛盾突出。主要是新增财源少，加之东庞煤矿透水减收、防治“非典”和政策性工资支出增加，导致可用财力减少，收支矛盾更加加剧。六是就业再就业压力大。全市需要就业人数90.2万人，新增就业岗位仅有9万个，供求之比高达1:10。七是经济发展环境有待改善。一些政府部门职能转变不到位，服务意识差，办事效率低，“四乱”现象仍未得到有效遏制。上述问题，已引起我们的高度重视，在新的一年里将下大力认真加以解决。

2004年主要工作及措施

2004年是实施“十五”计划的关键一年，也是推进跨越式发展、全面建设小康社会迈出新步伐的一年。我们要进一步增强加快发展的危机感和紧迫感，抢抓机遇，迎接挑战，求真务实，真抓实干，努力把改革发展推向一个新的高度。今年政府工作总体要求是：坚持以邓小平理论和“三个代表”重要思想为指导，深入贯彻党的十六届三中全会，省委六届三次、四次全会和市委六届四次、五次全会精神，继续高举发展大旗，弘扬主旋律，树立强烈的机遇观、科学的发展观、正确的政绩观，实施新型工业化、城镇化、农业产业化“三化联动”和科教兴市、开放带动战略，以结构调整为主线，继续抓好农业产业化、项目建设、民营经济、企业改革与壮大优势骨干企业、城镇建设五项重点工作，加快对外开放、服务业、培源壮财、社会保障、社会事业发展步伐，统筹经济与社会、城市与乡村、资源与环境和谐发展，推进跨越式发展和全面建设小康社会迈出新步伐。

今年主要预期目标是：国内生产总值增长12%以上，达到570亿元；城镇新增就业人数3.5万人，增长14%以上；城镇登记失业率控制在4%以内；全部财政收入增长11.5%以上，达到39.6亿元以上；全社会固定资产投资增长21%以上，达到260亿元以上；实际利用外资增长21.8%，达到7000万美元；城镇居民人均可支配收入和农民人均纯收入分别达到6580元和2860元，分别增长7%和5%；人口自然增长率为6.45‰;科技贡献率达到45%，提高2个百分点；高中段毛入学率达到5%，提高5.5个百分点；农村医疗保健和重大传染病预防工作实现新提高；空气质量二级或好于二级以上天数达到200天以上。

按照上述指导思想和目标要求，今年经济工作坚持牢牢把握“五个原则”：抢抓机遇推进跨越式发展的原则；全面、协调、可持续发展的原则；经济发展与群众生活协调发展的原则；实施“三化联动”优化城乡结构和区域经济结构的原则；发挥比较优势突出重点的原则。具体抓好“五个重点”，加快“五个步伐”。

抓好“五个重点”：

结构调整是经济工作的主线，必须把五项重点工作渗透到结构调整之中，强力推进，从根本上提高我市经济的整体水平和竞争力。

（一）以农民增收为核心，推进农业结构调整和产业化经营。认真落实今年中央1号文件和省委六届五次全会精神，高度重视“三农”问题，用新型工业化和城乡统筹的思路抓农业，力促农业增效和农民增收，实现县域经济的新跨越。一是调整结构增效益，进一步完善“五带十区”农业布局。全面、正确地贯彻农业结构战略性调整方针，按照“稳粮棉、强畜牧、优林果、扩蔬菜、扶特色”的思路，稳定和提高粮食生产能力，改善农产品品质，扩大专用粮种植面积，全年新增优质专用粮种植50万亩；围绕“五带十区”农业布局建设，做大做强畜牧、蔬菜、果品三大主导产业，培育发展食用菌、林木、牧草三个新兴产业。全年完成畜牧业增加值30亿元，占第一产业的比重达到37%，提高2个百分点；新增食用菌6.5万亩，设施蔬菜3万亩；新增用材林20万亩，完成太行山区优质干鲜果带建设7万亩。同时，积极推进农林复合经营，新增15万亩，提高土地产出率和农业综合效益。二是围绕“三化”抓联动，积极推进农业产业化经营。大力发展16个特色主导产业和龙型经济。重点抓好18个投资1500万元以上龙头项目，规范发展20个以上具有市场开拓和联结能力的农民专业合作经济组织。大力实施“农民培训”和“科技示范”两大工程，努力为农民进城务工和进入非农产业领域创造良好条件。全年农业产业化经营率达到57.8%，提高2个百分点。三是加强基础设施建设，提高农业综合生产能力。全力推进农业标准化生产，加强良种繁育、农产品生产管理标准、农产品质量检验监测、动植物保护和农业信息服务五个体系建设；大力推广节水和旱作农业技术，积极发展设施农业和生态农业；搞好太行山片麻岩的进一步开发和山区水土流失、黄河故道沙化土地综合治理，抓好国家、省级重点水利工程建设；进一步加大农业综合开发和扶贫开发力度，改善农村生产生活条件。四是深化农村改革，抓好农民减负增收。切实抓好中央、国务院出台的各项惠农政策落实，千方百计增加农民收入；坚持农村土地基本经营制度，实行严格的耕地保护制度；完善土地流转办法，发展适度规模经营；深化农村税费改革，取消农业特产税，降低农业税税率，进一步减轻农民负担；积极推进粮食流通体制改革，把对种粮农民的间接补贴改为直接补贴，提高种粮农民的积极性；深入开展专项治理，保护农民合法权益。全年全市农业增加值达到81亿元，增长4.5%。

（二）坚持以大投入带动大发展，实现项目建设新突破。继续把上项目、增投入作为经济工作的重中之重来抓，用大投入带动大调整，用大调整带动大发展。今年，全市共安排1000万元以上项目374个，总投资503.6亿元，年度投资143亿元。其中，投资10亿元以上项目10个。具体做到四个围绕：一是围绕优势产业上项目。如投资20.5亿元的宁晋纺织城，年度投资1亿元，三年内产值将达10亿元，实现利税1亿元。投资8.75亿元的鑫磊、邢矿、奎山水泥生产线，投产后全市可新增水泥产量450万吨。二是围绕优势骨干企业上项目。今年重点抓好以下“再造工程”：①依托邢钢优势产品，投资50多亿元，建设邢钢线材加工基地，经过3—5年努力，新增年销售收入100亿元，创利税10亿元，再造一个邢钢；②继续抓好邢矿非煤产业发展壮大，实现年销售收入50亿元，比上年翻一番多，再造一个邢矿；③华龙与新日清合作，投资6亿元改造提升，达到产值100亿元，再造一个华龙；④中旺与康师傅联合投资15亿元，到2007年产值达61亿元,再造两个中旺；⑤兴泰发电公司投资23亿元，新上2×30万千瓦供热机组，建成后可年发电31亿度，上缴税金1.5亿元，再造一个兴泰；⑥方圆集团投资8.5亿元加大技改力度，经过2—3年努力，使销售收入由4.2亿元提高到13亿元，再造一个方圆。三是围绕特色产业上项目。以研制开发微晶板材制品、太阳能级单晶硅切片、生物杀菌、酶制剂等技术为重点，培育一批高新技术产业和龙头企业。如投资2.6亿元的宁晋松宫单晶硅项目，将成为世界最大的单晶硅生产基地。四是围绕基础设施上项目。如总投资25.48亿元的邢临高速，长106公里，今年完成投资8亿元；总投资31.05亿元的青银高速公路邢台段，长117公里，今年完成投资9亿元；总投资4.39亿元的市区集中供热工程,今年完成投资1.2亿元。为确保项目工作落到实处，对投资10亿元以上和5000万元以上的项目，分别实行市、县领导分包责任制，继续下大力推进投融资体制改革。制定定资产投资项目核准制、备案制和配套政策，促进投资主体多元化；充分发挥股票、基金的融资功能，扩大发行企业债券；规范政府投资范围，加强对社会投资的引导，防止盲目和重复建设。继续加大项目谋划力度，争取我市更多产业项目和产品进入国家的“笼子”、省里的“盘子”。同时，认真落实我市《关于进一步优化经济发展环境的若干规定》，支持和保护项目建设和运营。对政府相关部门和人员设立四条“高压线”：①严禁滥设行政审批事项，刁难项目业主；②严禁乱检查和违法行政处罚；③严禁超标准、超范围收费；④严禁直接或变相吃、拿、卡、报，侵犯项目业主合法权益。凡触犯“高压线”的，不仅要追究当事人的责任，还要追究单位主要领导的责任。

（三）培育壮大特色产业，大力发展民营经济。坚持以结构调整为主线，力求把民营经济“规模做大、实力做强、质量做优”。全市民营经济实现增加值456亿元，实缴税金17.7亿元，均增长16%。一抓环境优服务。认真贯彻全市优化发展环境、扩大对外开放50万人广播电视大会精神，进一步清理和修订限制性的法规和政策，放宽市场准入，鼓励民营企业参与国有企业改组改制，允许非公有资本进入法律法规未禁入的基础设施、公用事业及其他行业和领域，并在投融资、税收、用地和外贸等方面与其他企业享受同等待遇；畅通政府与民营企业的沟通渠道，抓好涉企部门支持服务承诺的落实；完善中小企业贷款担保中心，探索多渠道融资方式和途径，着力解决制约民营经济发展的融资难、用地难、办事难等问题。二抓产业促升级。围绕产业升级、企业做大和提高产品市场竞争力，继续搞好产业结构、布局结构和产品结构调整，抓好农副产品深加工项目，推动特色产业快速健康发展；引导企业联合协作增股扩张，形成更大的骨干企业群；培育扶持产业龙头扩张升级，促进知名产业升级，一般产业晋档，新兴产业扩张。三抓园区上档次。搞好新建园区的科学规划和资本运营，加大招商引资和项目建设力度，大力发展科技型企业，引导区内企业实施品牌带动和网络营销战略，争创国家、省级示范区。四抓项目促发展。继续抓好509个投资500万元以上的项目建设，力争全年固定资产投入达到110亿元，增长20%以上，新增亿元以上项目30个，千万元以上项目250个。五抓机制求创新。引导大中型民营企业加快公司制改造，鼓励发展国家、集体、外商和个人多元投资的混合所有制经济，创新按劳分配与按生产要素分配相结合的分配制度，促进更多的资金、人才和科技成果向民营企业转移。

（四）进一步深化国企改革，走新型工业化之路。按照“工业立市”的要求，加快经济布局和结构调整，促进资金、人才、技术等生产要素向优势企业、优势行业聚集，着力构建以高新技术产业为先导、以主导产业为支撑、各行业协调发展的特色工业新格局，提高工业经济的整体素质和竞争能力。一是做大做强三大支柱产业和优势骨干企业。基础原材料以邢钢、邢矿、奎山、鑫磊为龙头，食品以华龙与新日清、中旺与康师傅联合企业为龙头，纺织以宁纺、方圆为龙头，进一步加速产业产品升级换代，提高经营规模和技术含量，抢占市场制高点，使“工业立市”建立在扎扎实实的基础之上。三大支柱产业产值占全市入统工业总量比重达到60%，提高3个百分点。对邢钢、邢矿、华龙、恒利等30家优势骨干企业，鼓励其以资本、品牌为纽带，抓改革、强管理、扩规模；其它优势骨干企业也要在强强联合、做大做强上做文章，加快发展步伐，使优势骨干企业年产值和利税分别占到全部入统企业的47%和55%。二是加大技术改造力度，增强企业核心竞争力。进一步加大用高新技术和先进适用技术改造传统工业力度，重点抓好中联鑫磊临城日产5000吨熟料回转窑生产线改造等40个续建技改项目和德龙中宽带钢生产线改造等160个拟新开工技改项目，全年完成技改投资38亿元，增长16%；80%的企业技术开发机构达到“四有”标准（有专门人员、稳定投入、试验手段、开发成果）。三是继续深化国企改革，下大力搞活亏困企业。以“两增、两减、两分”为主线，以产权制度改革为突破口，加快破产重组和国有资本退出步伐。市重点抓好防爆电机厂、薄板厂等10家市属国有企业的改组、改制和破产重组，积极稳妥地分离拖拉机厂、鲸鱼、中达等市属企业办社会职能，千方百计使“一拖两车”等几家亏损大户恢复生机，进一步搞活城市经济。同时，指导、协调好县（市、区）属国有企业改革，深化国有资产管理体制改革，完善国企改革配套政策。今年，各县市区属国有企业改革全部完成，市属国有企业改革完成80%。通过以上措施，全年实现入统工业增加值139亿元，增长14.5%；利税54.5亿元，增长15%。

（五）全力推进城镇建设，发挥对经济的拉动作用。紧紧围绕创建省级园林城市和历史文化名城大目标，按照促进城市产业形成，拉大城市框架，改善城市形象，便民利民和拉动城乡经济发展的原则，全力推进城镇建设。到年底，城镇化水平达到35.3%，提高2个百分点。着力在“一拓三高”上下功夫：一是拓展城市发展空间。坚持新区开发与旧城改造相结合，以新区开发为突破口，继续实施“市区北扩、东延、向西发展”方案，进一步搞好路网建设，拉大城市框架，完善和形成新的城市发展格局。二是高起点规划。全面修编市域城镇体系规划和市区总体规划，调整城市空间布局，强化城市规划管理，实现城市功能可持续发展。各县市区要按照“工业进园区、住宅进小区、物业进社区”的原则，走内涵式发展路子。三是高标准建设。全市完成城建总投资55亿元,其中市区37亿元，各县市18亿元。在市区，实施11项重点工程：⑴总投资18.7亿元，年度投资10.6亿元，完善市区路网；⑵总投资3.3亿元，年度投资1.8亿元，实施城市“四化”工程；⑶投资7700万元，对市中心区进行美化包装；⑷总投资5836万元，年度投资2276万元，治理小黄河下游和围寨河；⑸总投资28.8亿元，年度投资13亿元，实施供热供气供水工程；⑹总投资1.5亿元，年度投资1.3亿元，搞好污水处理厂续建和垃圾处理厂建设；⑺总投资2.2亿元，年度投资1.6亿元，建设城通信息管网和城区电网；⑻投资5450万元，搞好房地产交易中心和中心汽车站续建；⑼投资5亿元，开工建设住宅100万平方米；⑽投资3000万元，建设农贸市场7个；⑾投资2冈凳┏侵写甯脑旌途汕≌卫怼Ｔ谙爻钦颍灾氐阆厥械男〕钦蚪ㄉ栉黄瓶冢サ愦妫┐笸度耄忧砍钦蚧∩枋┙ㄉ瑁环植愦味酱僦傅际《ㄖ氐阏颉⒔ㄖ普蚪ㄉ瑁φ?—3年时间把莲子镇、河古庙等13个省定重点镇建成全省一流小城镇；指导好沙河市栾卸、内邱县岗底、邢台县前南峪等5个省定现代化试点村建设。四是高效能管理。牢固树立经营城市意识，加强土地资源经营，开放基础设施建设市场，加快市政公用事业改革，全面开放住房二级市场，最大限度地盘活经营性、商业性和收费性资产。同时，进一步深化城市管理体制改革，强化综合治理，全面提升城市管理水平和文明程度。在交通建设上，以搞好高速公路和农村公路建设为重点，全年投资23.5亿元，加快邢临高速一、二期和青银高速邢台段建设，完成106国道改建高速前期，加大邢临高速西进争跑力度；大力实施村村通民心工程,建设农村公路1126公里，使通油路村达到83%,提高23.2个百分点；搞好国省干线300公里公路建设和大中修任务，以更高的起点开创交通工作新局面。

加快“五个步伐”：

在跨越式发展和全面建设小康社会过程中，要始终坚持统筹兼顾，促进经济社会的协调发展。

（一）全方位扩大对外开放，加快招商引资步伐。进一步强化大开放意识，坚持“引进来”与“走出去”并举，外经、外贸、外资一齐上，努力打造多层次、宽领域、全方位的对外开放新格局。一是整合开放资源。对我市农业、矿产、特色主导产业、国有企业等资源进行整合，发挥整体优势，以引进跨国公司、外资大项目、国有企业改组改造和民营企业对外合作为重点，力争使一批有份量的重大项目落户邢台；努力扩大对内开放，在加强联合中求发展，在产业错位中求发展，在差别竞争中求发展。市高开区在扩区的基础上，加大投入，抓好启动十个片区、创引十个品牌、修建十条道路、办好十件实事等重点工程建设，成为我市对外开放的重要载体和“窗口”，全年实现主要经济指标增长50%以上，完成国内生产总值4亿元。二是优化出口结构和主体。实行市场多元化，调整出口商品结构，努力使外贸出口与产业发展战略有效对接。巩固扩大亚、欧、北美等传统市场，积极开拓俄罗斯、拉美和大洋洲等新兴市场。认真落实国家出口退税政策，抓好清河、南宫、宁晋等出口大县，邢钢、鲸鱼、晶牛等出口大企业，建材、羊绒及制品、单晶硅等大宗骨干出口商品，赋予更多的不同所有制企业外贸出口权，保持外贸出口的强劲增势。三是创新招商方式。把网上招商、 中介代理、以商引商同各类会议招商有机结合起来，实现招商方式新突破。进一步规范政务大厅，提高工作效率。主动接受京津沪等发达城市辐射，并紧紧抓住南资北上、产业北移机遇，适时在一些区域经济中心城市开展大型经贸洽谈活动。积极参与石家庄、邯郸、安阳等周边市打造冀南“经济发展带”和中原“经济圈”的合作，加快推进区域经济一体化。鼓励有条件的企业到上海、深圳和香港上市，拓宽筹融资渠道。鼓励更多的企业到境外投资办厂，带动劳务、技术、设备、材料和产品出口。同时，增加短缺原材料、关键技术和重大装备进口。全年引进省外资金23亿元，增长9.5%；出口创汇3.35亿美元，增长11%。

（二）围绕建设沟通晋冀鲁豫地区重要商贸物流中心，加快服务业发展步伐。立足我市区位优势，整合资源，扩大规模，提升档次，使服务业成为拉动城市经济和县域经济发展的重要力量。一是加快以旅游业为主的新兴服务业发展。打破区域界限，研究制定我市融入全省大旅游的规划方案，充分利用周边省市旅游资源，构筑以市区、邢台县、临城为中心的一日游或多日游黄金旅游线路；以开发利用自然资源为主，突出重点，打造一批以太行奇峡群、崆山白云洞等重点企业为龙头的特色精品项目；加强行业管理和基础设施建设，在重点景区搞好游、娱、购、吃、住、行一体的一条龙服务；开展旅游宣传和促销，增强对外吸引力。全年完成旅游业产值15亿元，增长50%。通过三年努力，使旅游业成为我市一个新兴产业和新的经济增长点，占GDP的比重达到5%以上。二是加快商贸流通企业改革和布局调整步伐。以建立健全统一开放、竞争有序的商品市场体系为目标，搞好市区商业网点建设规划，优化市场布局；进一步加快商贸企业改革步伐，改造提升传统服务业，完成市食品蔬菜集团等6家企业改制工作，争取市百货大楼等4家企业破产终结。各县市要进一步抓好特色专业市场建设和发展，大幅度提高聚集力和辐射力。全年医药业实现销售收入9.25亿元，上缴税金5600万元，均增长30%以上。三是大力发展现代流通业。积极推动连锁经营等现代营销方式，支持新亚、华天、华业、珂琳日化等龙头企业发展连锁经营和跨行业、跨所有制、跨区域的联合重组；鼓励有条件的连锁企业通过联合、资产重组、参股控股、特许经营等方式发展营销网络，促其扩张升级；大力推进现代物流业发展，进一步抓好糖酒、石油、烟草配送网络建设，使配送网点进一步向农村延伸。同时，适应社会需求和消费升级变化，积极培育发展住宅、汽车等消费热点和家政服务、社区服务、中介服务、信息咨询等新兴服务业，促进消费增长。全年第三产业实现增加值157亿元，增长12.5%。

（三）抓改革强征管，加快培源壮财步伐。一是坚持依法治税。严格执行《征管法》，实事求是地组织好财政收入，做到应收尽收，坚决杜绝虚收。二是深化财政改革。积极稳妥地推行规范的部门预算，继续深化预算管理、集中收付、政府采购和“收支两条线”改革，提高理财水平。进一步完善财政收入体制，促进县域经济快速发展。三是严格财政支出。坚持“有所为、有所不为”，优化支出结构，严格支出顺序，突出支出重点：①确保公教人员工资和机关正常运转资金需要。②加大改善农村生产生活条件和社会保障等事关人民群众生产生活的“民心工程”投入。③加大对农业、科技、教育、卫生等投入。④突出支持项目建设、国企改革、对外开放、民营经济等重点工作。⑤统筹安排社会事业，促进经济和社会协调发展。四是强化财政监督。硬化预算约束，加强对收刚魇栈亍⒅捶ú棵藕徒鹑凇⒅薪榈然沟募喽剑婪痘獠普诵星痹诜缦眨患岢智诩蟀煲磺惺乱担峋鲅顾跻磺胁缓侠淼目В喜屏Π齑笫隆＝鹑诠ぷ鳎鹑诨挂髡糯峁梗呕鹑诜瘢罅χС值胤骄梅⒄梗ψ龅健熬没睿鹑谛恕薄５侥甑祝迪执妗⒋钣喽罘直鸨饶瓿踉龀?3%和10%以上。

（四）抓好就业再就业工作，加快社会保障体系建设步伐。把扩大就业和提高社会保障能力放在更加突出的位置，持之以恒地抓好抓实。一是千方百计扩大就业。通过扶持搞活中小企业、大力发展三产、特色产业、民营经济和劳动密集型企业，为就业和再就业提供更多的岗位。抓好新上项目与再就业挂钩安置、买岗安置和以内部消化为重点的分流安置，强力推进就业再就业工作。认真抓好再就业和创业培训，引导下岗职工转变择业观念，培养和造就一批小企业创办者和自谋职业者。努力增加再就业资金投入，从今年开始，市县两级财政每年都要从预算资金中提取 不少于1%的专项资金用于促进就业和再就业。二是切实加强社会保障。按照先易后难、稳步推进的原则，继续依法扩大养老、医疗、失业保险覆盖面，强化社会保险资金征缴和监管，提高全市社会保障能力；继续把“低保”和“两个确保”作为社会保障的重中之重，做到应保尽保，足额发放；加快农村社会保障体系建设，探索建立农村养老保障体系，在有条件的地方建立农村最低生活保障制度。三是妥善解决好困难群众的实际问题。建立完善灾害救助、社会救济、社会互助工作体系和经常性工作机制，切实做好扶贫开发、救灾救济、拥军优属等工作；积极筹集救济资金，努力解决好贫困群众和各类弱势群体的生产和生活，保证群众有饭吃、有衣穿、有住处、能就医。

（五）坚持协调推进，加快社会事业发展步伐。一是大力推进科技创新。紧紧围绕“出成果、出人才、出生产力”的要求，继续深化科技体制改革，加快建立以企业为主体、产学研相结合的技术开发体系，完善科技服务体系；加快推进生物技术与新医药、新材料、光机电一体化、环保等领域的产业化项目实施，大力发展高新技术产业；加快科技兴农步伐，加强良种培育、节水灌溉等技术的研究和推广。全年完成国内先进水平科技成果35项，推广重大科技成果35项，主要农作物良种覆盖率达到98%。二是加快教育发展步伐。围绕全面贯彻党的教育方针和全面提高教育教学质量，把农村教育和高中阶段教育作为重中之重，统筹安排各级各类教育。深化教育改革，优化资源配置，以现代制造业、服务业技能型紧缺人才的培养培训和农村劳动力转移培训为重点，大力发展职业教育、成人教育和远程教育。实施高等教育大众化工程，构筑“两本三专”框架结构。加大教育投入，完善经费保护机制，全年新建校舍60万平方米，改造中小学危房4.8万平方米。三是实施人才兴市工程。树立人才是第一资源的观念，把人才工作摆在更加突出的位置，扎实抓好引才、育才、用才工作，营造尊重人才、鼓励创业的社会环境，形成人才脱颖而出、人尽其才的良好机制，努力为跨越式发展提供人才保证和智力支撑。四是加强公共卫生建设。以提高公共卫生服务水平和应急能力为重点，建立健全重大突发公共卫生事件的长效工作体系，全力做好“非典”、高致病性禽流感预防和农村卫生工作，提高疾病防治和突发公共卫生事件应急能力。继续推进医疗保险制度、医疗卫生体制和药品生产流通体制改革，整合城市医疗卫生资源，加快乡镇卫生院的布局调整和改造。以城市环境卫生和农村改水、改厕和除“四害”为重点，深入开展爱国卫生运动。五是走可持续发展道路。加强人口资源环境工作，促进人与自然和谐相处。做好基层基础工作，稳定低生育水平，提高人口素质；坚持资源可持续利用，依法保护和合理开发国土资源；继续实施环境保护“六项工程”，加大重点流域、重点城镇、重点区域的污染防治和清理整顿力度；大力抓好建筑节能，发展循环经济和清洁生产，促进各类社会资源的节约和综合利用。六是加强精神文明建设。广泛开展爱国主义、社会主义、集体主义教育，大力弘扬和培育民族精神。认真落实《公民道德建设实施纲要》，开展形式多样的宣传教育和道德实践活动，推动文明城市、文明行业、文明机关、文明生态村建设，努力提高社会文明程度。深入开展创建学习型邢台活动，促进人的全面发展。围绕建设文化大市，大力加强文化体育建设，提高人民群众的文化素质和健康水平。七是增强全民国防意识。继续把“双三好”活动引向深入，支持驻邢解放军、武警部队和民兵预备役建设，切实解决好复员转业退伍军人的安置问题。依法管理宗教事务。统计、民政、外事、侨务、档案、妇女、儿童、残疾人、地方志、无线电管理等各项工作都要有新的进步。

切实加强和改进政府工作

适应新形势、新任务的要求，认真践行“三个代表”重要思想，切实加强和改进政府工作，推进政府职能转变和管理创新，树立“为民、务实、清廉”的良好形象。

（一）与时俱进，开拓创新。按照胡锦涛总书记“欠发达地区要实现跨越式发展，更需要大力发扬与时俱进、开拓创新精神”的要求，大力弘扬“树正气、讲团结、求发展”主旋律，牢固树立强烈的机遇观、科学的发展观、正确的政绩观，坚决克服思想和体制“两大障碍”，始终保持良好的精神状态，在解放思想中统一思想，在与时俱进中加快发展，真正加深对各项事业发展规律的认识，真正理出一条符合实际的工作思路，真正拿出一些解决问题的有效办法，努力开创各项工作新局面。

（二）执政为民，心系民生。各级政府组成人员都要站在执政为民的高度，把实现人民的愿望，满足人民的需要，维护人民的利益，作为政府工作的根本出发点和落脚点。按照省委、省政府统一部署，今年要认真实施好“民心工程”，突出办好就业再就业、农民增收、解决人畜饮水困难、中小学危房改造和新建校舍、重大疫病防控体系建设、社会救济、城乡公路建设、城镇环境改善、文化体育设施建设、社会治安等十项实事。以上十项实事，事关人民群众最现实、最关心、最直接的利益，要抓好责任分解，保证资金投入，搞好公开监督，确保工作落实，使这些实事真正成为振奋人心、凝聚民心的鱼水工程。

（三）转变职能，优化环境。以这次机构改革为契机，推进政企、政资、政社分开，把政府不该管的事交给企业、市场、社会组织和中介机构，努力解决政府职能越位、缺位和错位问题，切实把各级领导的精力更多地放在推进社会发展和解决民生问题上，把政府经济管理职能转移到主要为市场主体服务和创造良好发展环境上来。继续深化行政审批制度改革，落实好省、市出台的各项优惠政策；全面推行政务公开，实行岗位轮换制，坚决制止“四乱”行为，提高行政服务质量和效率；完善社会信用体系，努力营造亲商、重商、扶商、护商的良好氛围；下大力搞好“四五”普法，规范执法行为，完善村民和社区居民自治，保证人民群众依法行使民主权利。

（四）求真务实,真抓实干。坚持实事求是的思想路线，大力发扬“严细深实快”五字作风，出实招，办实事，求实效；经常深入基层，深入群众，切实解决好改革发展稳定中的重大问题和群众生产生活上的紧迫问题；下大力转变工作作风，扑下身子真抓实干，压缩会议、减少应酬，集中精力谋大事、干大事；坚决反对形式主义和官僚主义，以实际行动赢得人民群众信赖和支持。

（五）强化措施，维护稳定。牢固树立积极主动的稳定观，正确处理改革、发展与稳定的关系。继续加强社会治安防范体系建设，惩治“法轮功”等邪教组织，严厉打击各种刑事犯罪活动。在全市农村深入开展“讲科学、讲文明，全面建设小康社会”教育活动。加强信访工作，妥善调解矛盾，力求把问题解决在基层，化解在萌芽状态。同时，下大力整顿和规范市场经济秩序，切实抓好安全生产，为改革发展创造良好社会环境。

（六）依法行政，廉洁勤政。认真贯彻《行政许可法》，把政府权力严格限制在法律范围之内，防止权力滥用；牢固树立马克思主义的世界观、人生观、价值观和正确的权力观、地位观、利益观，坚持不懈地抓好廉洁自律、查处大案要案、纠正部门和行业不正之风三项重点工作；严格执行述职述廉、重大事项报告和民主评议制度，自觉接受人大、政协监督，加强组织监督和社会监督，不断改进政府工作，更好地完成人民赋予我们的光荣使命。

各位代表！面对新的机遇和挑战，我们要坚持以邓小平理论和“三个代表”重要思想为指导，在中共邢台市委的领导下，继续高举发展大旗，弘扬主旋律，进一步解放思想，开阔眼界，抢抓机遇，迎接挑战，求真务实，真抓实干，为推进全市跨越式发展取得新进展、全面建设小康社会迈出新步伐而共同奋斗！
